# The Intersection of Oncology Prognosis and Cancer Rehabilitation

**DOI:** 10.1007/s40141-017-0150-0

**Published:** 2017-03-01

**Authors:** Sean Robinson Smith, Jasmine Yiqian Zheng

**Affiliations:** Department of Physical Medicine & Rehabilitation, University of Michigan, 325 E Eisenhower Pkwy, Ste 100, Ann Arbor, MI 48108 USA

**Keywords:** Cancer rehabilitation, Cancer prognosis, Prognosis and supportive care, Prognosis and rehabilitation, Geriatric cancer rehabilitation, Cancer rehabilitation attitudes

## Abstract

**Purpose of review:**

This review examines the delivery of rehabilitation care to cancer patients with relation to disease prognosis. This includes the evaluation when patients are referred for rehabilitation services and the effectiveness of rehabilitation interventions across the cancer continuum.

**Recent findings:**

Although prognosticating life expectancy is difficult, referrals for rehabilitation interventions appear to be affected by physician attitudes towards patients with advanced disease, in part because of misconceptions about the nature of rehabilitation for oncology patients. Rehabilitation may also be underutilized in long-term survivors with no evidence of disease. Despite this, our review found that rehabilitation in advanced disease, end-of-life, geriatric cancer patients, and in long-term survivors can be beneficial. There is a relative dearth in studies on rehabilitation interventions specifically at the end-of-life.

**Summary:**

Cancer rehabilitation can be helpful to patients along the spectrum of cancer prognoses. Examining more accurate ways to prognosticate life expectancy, improving communication and education between oncologists and rehabilitation team members, and modifying survivorship plans to include patient education on functional changes over time may improve the delivery of rehabilitation care.

## Introduction

The dynamic and complex nature of oncology patients creates challenges in providing rehabilitation care. As patients progress through treatments and/or their disease advances, the rehabilitation target may move—a goal of independent functioning and returning to work may abruptly change to requiring assistance with activities of daily living and moving to an accessible home. These changes are even more pronounced when a patient’s prognosis changes, as someone who is expected to be a long-term survivor of cancer will undoubtedly have different rehabilitation goals and expectations than someone with a life expectancy fewer than 6 months.

The evolution of symptom burden and impaired function as the disease trajectory advances is an ongoing area of study, but it is clear that the rehabilitation needs of patients change as their prognosis does and that all patients across the cancer continuum potentially have rehabilitation needs. For example, Beernaert et al. found that patients with advanced cancer report poorer function and increased fatigue and pain compared to other cancer patients after controlling for age, gender, and type of cancer [1]. Additionally, patients undergoing initial cancer treatment with curative intent also had more severe pain and worse physical function than healthy controls.

We sought to evaluate prognosis as it intersects with delivering rehabilitation and supportive care services. Using the search terms listed in Table 1, we found a variety of studies evaluating the effectiveness of rehabilitation across the spectrum of prognosis associated with cancer, though interventions were often not compared between cohorts with different prognoses. Many variables confound the evaluation and delivery of rehabilitation services across the cancer continuum, including clinician bias with respect to prognosis, supportive care needs, difficulty predicting prognosis, and inadequate care delivery models. In this review, we will discuss these components vis-à-vis the relationship between prognosis and cancer rehabilitation, as well as current relevant studies evaluating rehabilitation across the cancer continuum.

**Table 1:** Search terms used for data collection for this article

“Cancer prognosis and rehabilitation”^a^	
“Cancer survivorship and rehabilitation”	
“Long-term survivors and cancer and rehabilitation”	
“Cancer rehabilitation and falls”	
“Geriatric and cancer rehabilitation”	
“Prognosis and symptom management or rehabilitation or palliative care”	
“Cancer and function and rehabilitation”	
“Palliative care and rehabilitation and prognosis”	

^a^Most helpful search term

### Physician Attitudes About Rehabilitation in Different Stages of Disease

Despite efforts to improve patient access to rehabilitation and supportive services, physician bias regarding rehabilitation across the spectrum of disease may lead to unmet patient needs. Spill et al. found that only 8% of the medical oncologists—compared to 35% of physiatrists—would recommend a patient with functional goals and advanced cancer (6–12 months of estimated survival) be admitted to an inpatient rehabilitation unit, but 70% of the combined cohorts believed that a patient must fully understand their prognosis before entering into a comprehensive rehabilitation program [2]. It is worth pointing out that while physiatrists in general were more willing to accept a patient with advanced cancer to inpatient rehabilitation, nearly two-thirds of those surveyed did not think that a patient with 12 months or less estimated survival should undergo comprehensive rehabilitation. Another study found that palliative care physicians would like to better incorporate rehabilitation in end-of-life care, but when surveyed, they reported concerns that rehabilitation would provide false hope to patients and that a palliative care service lacks the knowledge and resources to adequately carry out high-quality rehabilitation measures [3••]. This disconnect may be due to perceptions that rehabilitation need be a lengthy process that will take up a significant proportion of the patient’s remaining life expectancy. Table 2 outlines physician attitudes that may be barriers to referral to rehabilitation services in cancer patients.

**Table 2:** Physician barriers to rehabilitation referrals

Study	Barrier to rehabilitation referral
Spill [2]	Poor use of patient time and medical resources at end-of-life, sometimes unnecessary
Runacres [3••]	Inadequate training to provide rehabilitation, do not want to give the patient false hope at end-of-life
Virgo [74]	Inadequate awareness and training to evaluate for rehabilitation needs, defensive medicine
Libert [75]	Inadequate training to screen for symptoms, insufficient clinic time
Cheville [76]	Lack of recognition and documentation of functional deficits

In fact, cancer rehabilitation, when focusing on addressing specific impairments, may be quite efficient and is effective in reducing symptom burden [4]. This bias against rehabilitation in end-of-life scenarios, however, may create imbalanced care models and lead to patients being undertreated. For physiatrists, those who specialize in cancer rehabilitation and therefore are more comfortable managing patients with advanced cancer may be more aggressive in developing rehabilitation plans. Furthermore, it has been shown that medical oncologists who are comfortable with end-of-life care are far more likely to refer for palliative—but not necessarily end-of-life—services [5].

Physician attitudes about prognosis also appear to be the most important consideration in initiating palliative systemic treatment in advanced cancer, as Buiting et al. found that nearly half of medical oncologists documented it as part of their decision making [6]. Performance status, on the other hand, was only considered in 16% of decisions to provide systemic treatment, underscoring that life expectancy is often considered to be more important than physical function when evaluating whether or not to aggressively treat patients.

In an attempt to standardize when patients are referred for rehabilitation based on prognosis, Jarlbaek et al. suggest that patients who live longer than 2 years post-cancer diagnosis may be better candidates for rehabilitation programs than hospice and end-of-life care programs. In their study of over 24,000 patients, 78% who died did so within 2 years of cancer diagnosis, and many of those that lived longer than 2 years often lived for several more years [7]. This study does not control for many factors, including cancer diagnosis, and does not look at the specific rehabilitation needs of patients before determining whether or not rehabilitation would have been warranted. For example, a patient who lives 18 months after cancer diagnosis may derive benefit from a short inpatient rehabilitation stay if functional impairment is present early on, or a patient with 3 months to live may have less pain secondary to rehabilitation interventions.

### Challenges in Prognosticating Life Expectancy

It is not clear that providers can accurately predict prognosis. This is particularly worrisome if physician bias about perceived prognosis is a major factor in determining who receives rehabilitation care. A recent study found that using an assessment tool—the palliative prognostic score (PaP)—was significantly more accurate in determining both 30- and 100-day survival when compared to using the tool combined with clinician prediction, and to clinician assessment alone [8]. Other studies have also shown objective assessment to be more accurate than clinician gestalt regarding prognosis, particularly when prognosticating 3 months or less into the future [9].

Rehabilitation may be time consuming, and oncologists may be biased against referring patients with limited life expectancies to these services. Because of this, it is important to factor in patients’ goals of care; for example, some patients with less than 3 months to live may desire to undergo 2 weeks of inpatient rehabilitation if it reduces caregiver burden. Unfortunately, patients are generally unsure of their prognosis and tend to be more optimistic than oncologists, as demonstrated in a recent study that found 68% of the patients to be discordant with their oncologists when estimating life expectancy [10]. Another study showed that discordance also exists between physicians and surrogate decision makers of patients in 53% of the patients and that factors contributing to this difference included the need to be feel optimistic to benefit the patient, religious beliefs, and lack of physician understanding about a patients’ ability to recover from physical and cognitive impairments [11].

Unrealistic expectations about prognosis have the potential to create unrealistic goals of care. This underscores the need for effective communication between patients and their oncology teams about long-term planning, including rehabilitation care. Conversely, patients often experience hope and optimism despite understanding their life expectancy. Reasons for this are multifactorial and not always well-understood by health professionals but may explain why patients seek restorative interventions such as cancer rehabilitation despite a poor prognosis [12].

### Delivering Rehabilitation and Supportive Care Across the Continuum

Understanding when to refer patients for rehabilitation services presents an ongoing challenge. Numerous organizations have advocated and developed criteria for evaluation of patients for rehabilitation needs and other supportive care services without a qualifier of patient prognosis [13–15]. The message is clear: all patients should be evaluated for and provided access to rehabilitation treatment regardless of when their cancer treatment began or their estimated life expectancy.

For patients in whom the disease is presumably cured, the Commission on Cancer (COC) has attempted to improve evaluation and referrals for long-term care by mandating survivorship care plans (SCP). These serve as a way to inform patients about potential short- and long-term complications from cancer or its treatment, and explain how to obtain supportive care services [16]. Unfortunately, while SCPs increase the likelihood of patients attending follow-up visits, they do not appear to increase awareness about the late or long-term effects of cancer [17], and there is a lack of consensus on format and delivery of the plans [18].

Furthermore, SCPs provide an early opportunity to inform patients about potential rehabilitation needs, but they are not designed for patients with advanced cancer or to inform patients about functional decline as disease advances and prognosis changes. Although plans of care in advanced cancer have been proposed, there exists no standard in providing patients information about symptom management and rehabilitation need [19].

For patients with advanced cancer or higher symptom burden, the National Comprehensive Cancer Network (NCCN) has proposed criteria to determine if patients should be referred for palliative care services in order to provide symptom management. Specifically, the NCCN recommends that if patients display any one of the following six attributes, palliative care referral should be initiated: (1) uncontrolled symptoms; (2) moderate-to-severe distress; (3) serious comorbid physical or psychosocial condition; (4) advanced or progressive disease without available curative therapy; (5) patient/family concerns about decision making; (6) or patient/family request for palliative care [20]. Levy et al. found that referring to palliative care based on these criteria led to reduced symptom burden and more patient referrals [21]. Numerous studies have shown that early palliative care reduces symptom burden [22, 23], but care delivery is inadequate for reasons that include a lack of personnel and inefficient and inconsistent referral mechanisms [24]. For example, one retrospective review of 100 patients at the MD Anderson Cancer Center found that patients referred early for supportive care may be those who are younger and have obvious treatment-related side effects and that radiation oncologists were more likely to refer than medical oncologists [25]. It is important to note that these studies show that early intervention reduces symptom burden, which has not been specifically studied in the context of cancer rehabilitation.

A recent meta-analysis of outpatient palliative care referrals found inconsistent evaluation and referral mechanisms for patients across the cancer continuum. The most important criteria for referring a patient were physical symptoms (62% of the studies in the analysis) and cancer trajectory (62%), followed by prognosis (33%) and performance status (33%) [26••]. The analysis concluded that there was inconsistency in cutoffs of symptom assessment tools and timing for referrals and that better standardization of the process would improve identification of supportive care needs of patients.

### Rehabilitation in Advanced Cancer and End of Life

As outlined above, uncertainty about the benefits and necessity of rehabilitation in cancer care largely center on patients with a poor prognosis. This may be due to multiple factors, including considerations surrounding the appropriateness of time- and resource-intensive services for patients with limited life expectancy. While palliative care and hospice at the end-of-life is generally felt to be important and effective [27, 28], cancer rehabilitation is not as widely adopted nor understood in this patient population and may be underutilized [29].

Rehabilitation at the end of life may cover many facets, including outpatient, inpatient, and hospice-based interventions. Outpatient intervention has the potential to provide rapid assessment and relief of symptoms. Referral to a physiatrist to diagnose and treat neuromusculoskeletal, pain, functional, and other symptoms should be considered as a part of impairment-driven rehabilitation [4]. Many patients with advanced disease seen in a cancer rehabilitation clinic will have metastases to the bone and/or brain, and physiatrists are well-equipped to intervene in both scenarios.

Patients with bony metastases may need expert assessment of lesions to determine stability and proper management. For example, vertebral metastases often do not require surgical or radiation-based intervention, though this must be first evaluated systematically based on the location, size, and nature of each lesion [30]. Physiatrists are often key members of the multidisciplinary spine oncology team, both in providing this assessment and the subsequent treatment, including bracing, exercise, medications, and interventional pain procedures [31]. Furthermore, rehabilitation and resistance training concurrent with radiation in the presence of axial and peripheral bone metastases has been shown to improve bone density and strength without contributing to fractures [32].

Central nervous system lesions also often require physiatric evaluation and management, as primary and metastatic brain tumors can create numerous cognitive and neurologic deficits. Despite the ultimately poor prognosis of these cancers, rehabilitation intervention can improve function and quality of life, safety and independence, and reduce resource utilization by improving care coordination and preventing injury. Difficulties in accurately prognosticating survival in the presence of brain metastases has the potential to reduced appropriate rehabilitation utilization. One study of 2700 cases found that 45% of the estimations were off by 6 or more months, and 18% were off by 12 months. Performance status—which rehabilitation may improve—and age may be the biggest factors in determining prognosis in this patient population [33••].

Rehabilitation in the setting of brain metastases involves managing the effects of both the tumor(s) and the surgery and/or radiation. Whole brain radiation, for multiple or large lesions, can cause significant long-term cognitive impairment but is becoming less common with the advent of more precise radiation-based interventions [34]. Risk factors for radiation-induced cognitive impairment include higher total and fractionated dosing, increased brain volume radiated, combined chemotherapy, age over 60 years or less than 7 years old, and premorbid vascular disease [35]. Cognitive rehabilitation may take many forms and has been shown to improve executive functioning, working memory, and other areas of cognition to varying degrees [36] despite a Cochrane review of cognitive rehabilitation in brain metastases demonstrating no strong evidence to support any one specific non-pharmacologic intervention [37]. Therefore, treatment should be supportive and multidisciplinary, tailoring to each patient’s needs based on their symptoms. In addition to cognitive rehabilitation, this may include pharmacologic management by a physiatrist and interventions to boost physical activity.

Regardless of the presence of metastases, inpatient rehabilitation improves function and is safe in patients with advanced cancer. Although patients with advanced cancer may utilize inpatient rehabilitation more than those with controlled disease [38], Sliwa et al. found that patients with metastases benefitted from inpatient rehabilitation similarly to patients without metastatic or advanced disease, regardless of the presence of common inpatient rehabilitation diagnoses such as spinal cord injury (SCI), stroke, or neuromuscular disease [39••]. Furthermore, patients with impairments such as SCI due to tumor compression have been shown to benefit from inpatient rehabilitation [40]. One study comparing 143 patients with malignant spinal cord compression found that patients with cancer-related SCI had similar length of stay, functional scores, and discharge location compared to non-traumatic SCI patients without cancer [41]. Inpatient rehabilitation has been consistently shown to improve function and help ensure a safe discharge home in many other advanced and complex cancer patient populations [42], including brain tumor [43], hematologic malignancy [44], paraneoplastic disease [45, 46], and stage II–III tumors [47]. The benefits of inpatient rehabilitation in cancer patients also appear to be independent of age [48].

In the end-of-life and hospice setting, rehabilitation interventions have been shown to improve independence, reduce anxiety and depression, and improve fatigue, among other benefits [49–52]. One randomized control trial compared hospice patients who received daily rehabilitation compared to usual care, and found that the rehabilitation group resulted in less unmet needs at the end of life in psychological, physical, activities of daily living, care and support, and pain domains. The rehabilitation intervention group also utilized fewer resources [53••]. Furthermore, studies have shown that musculoskeletal pain is prevalent at the end of life [54], and the rise of ultrasound-guided procedures is allowing more rapid intervention for acute pain during hospice, opening avenues for physiatric management of end-of-life pain [55].

Finally, it is important to discuss the rehabilitation of geriatric patients with cancer. Even when the cancer is early stage, older patients are at a greater risk for morbidity and mortality, in part due to cancer treatment accelerating the aging process, and goal setting must be done in the setting of both the disease and the patient’s age [56]. Nevertheless, older adults with cancer have rehabilitation and supportive care needs across the continuum and should not be overlooked despite what may seem like a poorer prognosis than younger disease-matched patients [57]. In fact, Pergolotti et al. found that there are numerous potentially modifiable functional deficits in older adults with cancer, but that even when impairments are identified, rehabilitation services are under-utilized [58]. This is a larger problem when one considers that over 60% of pain in geriatric patients at the end of life is musculoskeletal and that rehabilitation interventions are uniquely suited to address these symptoms [59].

Another important area of rehabilitation concern is fall risk in the geriatric cancer patient. Spoelstra et al. found that older cancer survivors are 16% more likely to experience a fall than their cancer-free peers [60••], which may be due to multiple factors, including chemotherapy-induced peripheral neuropathy, deconditioning, and cognitive impairment [61]. Rehabilitation interventions to improve balance, endurance, strength, and to provide adaptive equipment and pain relief all can decrease the risk of falls in this population. Studies have shown that geriatric patient independence with ADLs is essential to quality of life and survival [62] and that rehabilitation interventions are crucial to preventing functional decline [63]. Despite this, rehabilitation needs in geriatric cancer patients may be overlooked despite their requiring and benefitting from rehabilitation services more than other patient cohorts.

### Rehabilitation in Long-Term Survivors and Those with a Favorable Prognosis

Although much of the controversy about the efficacy and appropriateness of rehabilitation understandably centers on the end of life, it is important to note that patients with the opposite prognosis—those whose cancers have been cured and/or who are expected to have a normal lifespan—may have rehabilitation needs overlooked, because lifesaving treatment has completed, and it is presumed that patients will recover from deficits over time. This is not always the case, as one study found that 38.2% of the unmet needs in cancer survivors 2-, 5-, and 10-year post-treatment were physical needs [64]. The long-term negative effects of cancer and its treatment have been well documented in the literature and are at the core of the cancer rehabilitation specialty [65]. Cancer care guidelines consistently recommend long-term, longitudinal assessment of supportive care needs, including rehabilitation [13–15].

The decline in function related to cancer treatment occurs early. For example, breast cancer patients experience increased fatigue and lower physical function and activity during and after chemotherapy, neither of which improve significantly without intervention [66]. Additionally, patients self-report a lower quality of life during the first year of being disease free compared to their perception of quality of life prior to disease onset, as well as compared to 5-years after being diagnosed with cancer [67]. This underscores the importance of early intervention and for the rest of the cancer survivor’s life. Different models have been proposed for this, including the prospective surveillance model, in which providers follow patients from diagnosis, and through both oncologic treatment and rehabilitation [68, 69]. Depending on the patient population, different approaches may be necessary, such as an interdisciplinary team that addresses the challenges faced by pediatric cancer survivors transitioning to adulthood [70].

While rehabilitation and exercise have consistently been shown to be beneficial across the cancer continuum including in long-term survivors [71, 72, 73], it is unclear which specialty and care delivery model is best equipped to meet the challenges of the growing cancer survivor population. Primary care has taken up the mantle of managing deleterious effects of cancer—the American Society of Clinical Oncology’s Survivorship Symposium is a joint effort between medical oncology and primary care—despite the fact that primary care physicians and oncologists both self-report not having the proper training, nor the in-clinic time, to evaluate and treat the physical and cognitive sequelae or cancer treatment [74, 75]. Furthermore, when rehabilitation needs are diagnosed, referrals for rehabilitation services are lacking [76–78].

Physiatrists and other rehabilitation providers are uniquely suited to manage neuromusculoskeletal and cognitive deficits in cancer survivors, and their involvement may reduce unmet rehabilitation needs in this population [16]. Improving function becomes all the more important when one considers that weight gain is a risk factor for cancer recurrence among those with no evidence of disease [79]. As the number of cancer survivors and therefore study cohorts grow, research may help determine the optimal pathway to long-term recovery; until then, it is clear that patients must take ownership of their care and empower themselves with all available resources [80].

Finally, rehabilitation has been consistently shown to help cancer survivors return to work, which is beneficial both economically and for patient well-being, as cancer survivors who have job-loss related to a health issue have lower health-related quality of life [81]. Fortunately, patients with support from rehabilitation physicians, and their family and friends, have not only been shown to be more likely to return to work but also to have a positive outlook on their employment despite a potential change in function caused by cancer [82, 83]. In one study looking at high-intensity training in long-term cancer survivors, rehabilitation interventions made it easier to return to work [84]. A comprehensive, multidisciplinary approach may be needed to help patients with no evidence of disease return to work, particularly in patients with negative predictors of returning to work, including older age, lower income, history of chemotherapy, a job requiring heavy physical activity, and low education level [85].

## Conclusion

A patient’s medical prognosis has the ability to impact rehabilitation and supportive measures in many ways, including physician bias and resultant lack of proper referrals. Despite this, rehabilitation interventions appear to be generally effective in improving function and reducing symptom burden in cancer patients across the spectrum of different prognoses. Unfortunately, studies evaluating interventions at the end of life are limited, and more likely to be short series or case reports, making the literature imbalanced in favor of interventions in the earlier stages of disease [86]. This creates potential physician bias and may explain some perceptions of rehabilitation in advanced disease.

Additionally, most of the available research focuses on palliative care, which can include management of symptoms treated by rehabilitation providers, such as pain and functional impairment, but is a broad topic, and the studies are often nonspecific about what palliative care interventions are offered. Although there is overlap between cancer rehabilitation and palliative care, it is important to note that there are distinctions in the two fields, primarily that palliative care typically addresses end of life considerations in more than just cancer patients—including spiritual and social or financial concerns—and cancer rehabilitation emphasizes restoring function and reducing symptom burden through identification and management of specific impairments in cancer patients [29].

It is important that rehabilitation be considered in all cancer patients who require it, regardless of prognosis. The challenge facing clinicians and researchers is developing actionable rehabilitation referral mechanisms and interventions specifically tailored to patients at different points of the disease continuum.
